# Supplementary material for: Genetic Deletion of DNAJB3 Using CRISPR-Cas9, Produced Discordant Phenotypes
Source: Genes (Basel). 2023 Sep 24;14(10):1857. doi: 10.3390/genes14101857 (PMC10606339; doi:10.3390/genes14101857)
Supplement: Supplementary file 1 [file genes-14-01857-s001.zip › genes-2592318-supplementary.pdf]

SUPPLEMENTARY MATERIALS

Genetic Deletion of DNAJB3 Using CRISPR-Cas-9, -Produced Discordant Phenotypes

Nejat et al., Genes, 2023

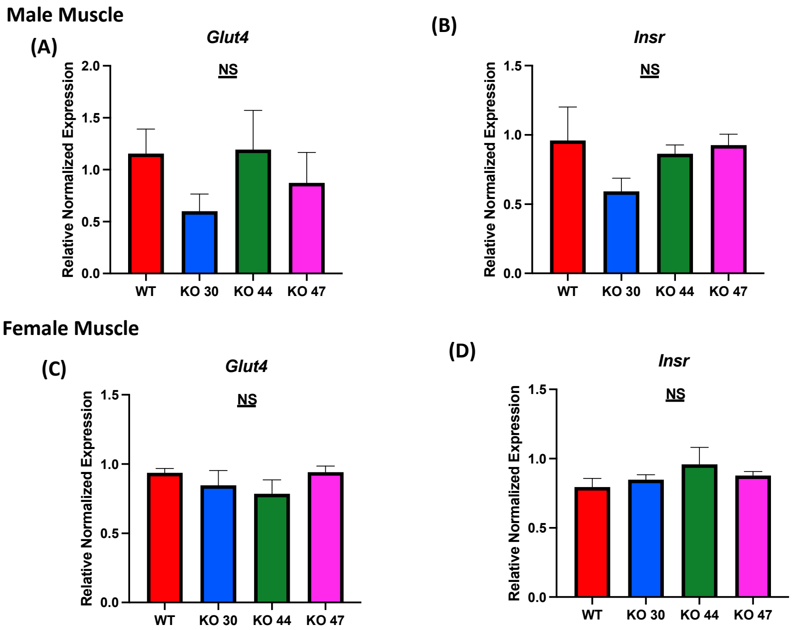

Figure S1. Role of DNAJB3 on regulating glucose metabolism in muscle of diet induced obese male and female mice. (A,C) mRNA levels of Glucose Transporter Type 4 (Glut4) in muscle tissue of male and female mice. **(B,D)** mRNA levels of Insulin Receptor (Insr) in muscle tissue of male and female mice. *An asterisk indicates significance compared to WT, while NS indicates no statistical significance across groups or compared to WT.. Data is presented as mean ± SEM (n=6). P <0.05.*

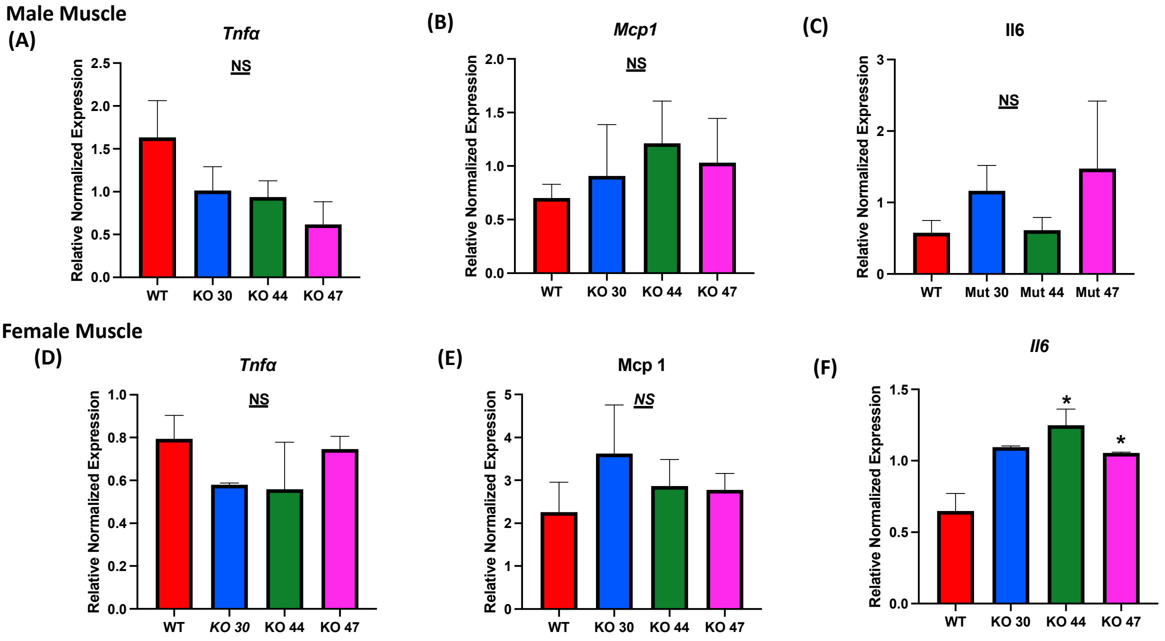

Figure S2. Role of DNAJB3 on inflammation in the muscle of diet induced obese male and female mice. (A, D) mRNA levels of tumor necrosis factor a (Tnfa) in muscle tissue of male and female mice. **(B, E)** mRNA levels of monocyte chemoattractant protein1 (Mcp1) in muscle tissue of male and female mice. **(C, F)** mRNA levels of Interleukin 6 (Il6), in muscle tissue of male and female mice. An asterisk indicates significance compared to WT, while NS indicates no statistical significance across groups or compared to WT. *Data is presented as mean ± SEM (n=6). P <0.05.*

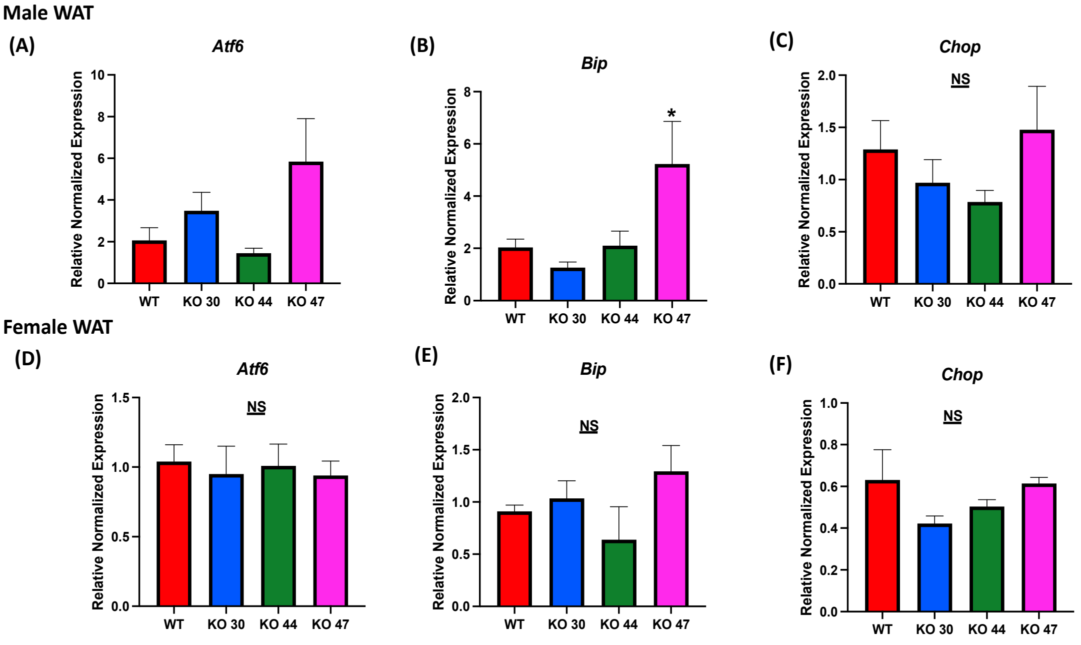

Figure S3. Role of DNAJB3 on ER stress in the adipose tissue of diet induced obese male and female mice. (A, D) mRNA levels of Activating Transcription Factor 6 (Atf6) in white adipose tissue of male and female mice. **(B, E)** mRNA levels of Binding Immunoglobulin Protein (Bip), in white adipose tissue of male

and female mice. **(C, F)** mRNA levels of C/EBP Homologous Protein (Chop) in white adipose tissue of male and female mice. An asterisk indicates significance compared to WT, while NS indicates no statistical significance across groups or compared to WT. *Data is presented as mean ± SEM (n=6). p <0.05.*

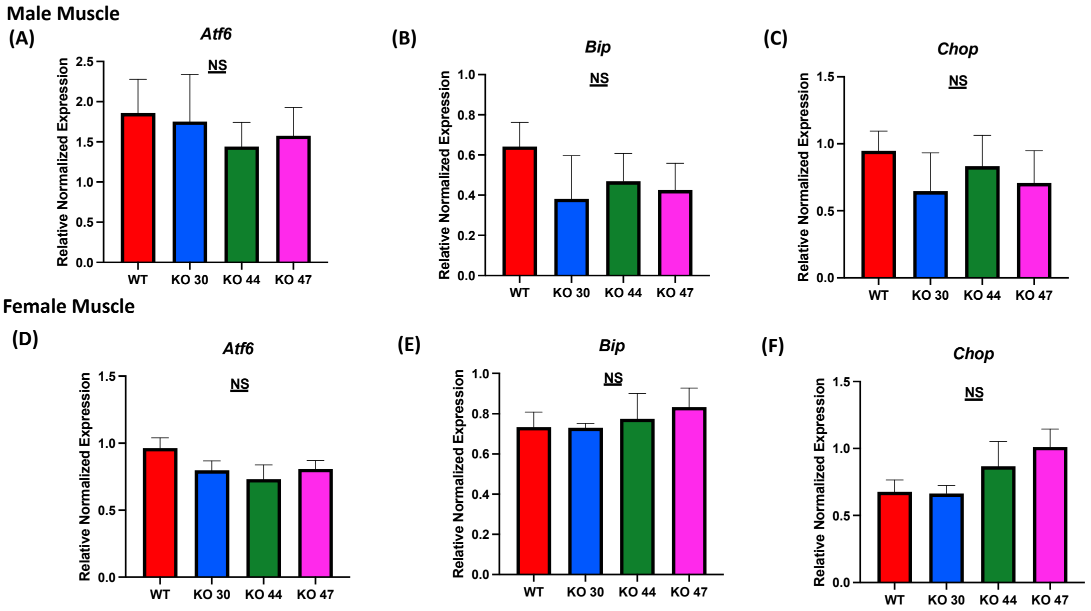

Figure S4. Role of DNAJB3 on ER stress in the muscle diet induced obese male and female mice. (A, D) mRNA levels of Activating Transcription Factor 6 (Atf6) in muscle tissue of male and female mice. **(B, E)** mRNA levels of Binding Immunoglobulin Protein (Bip) in muscle tissue of male and female mice. **(C, F)** mRNA levels of C/EBP Homologous Protein (Chop) in muscle tissue of male and female mice. An asterisk indicates significance compared to WT, while NS indicates no statistical significance across groups or compared to WT. *Data is presented as mean ± SEM (n=6). p <0.05.*
